# Plasma Tissue Factor Pathway Inhibitor Levels Correlate with Disease Activity and Are Associated with Altered Thrombin Generation in Pediatric Inflammatory Bowel Disease

**DOI:** 10.3390/ph19050738

**Published:** 2026-05-08

**Authors:** Alexander Meyer, Benno Kohlmaier, Theresa Bauer, Siegfried Gallistl, Wolfgang Muntean, Barbara Silbernagel, Harald Haidl, Axel Schlagenhauf

**Affiliations:** 1 Division of General Pediatrics, Department of Pediatrics and Adolescent Medicine, Medical University of Graz, 8036 Graz, Austria; 2 Division of Haematology, Department of Internal Medicine, Medical University of Graz, 8036 Graz, Austria

**Keywords:** pediatric inflammatory bowel disease, tissue factor pathway inhibitor, thrombin generation, Crohn’s disease, ulcerative colitis

## Abstract

**Background:** Patients with inflammatory bowel disease (IBD) exhibit a hypercoagulable state with increased thrombotic risk. Previous studies demonstrated elevated thrombin generation in pediatric IBD, paradoxically accompanied by prolonged lag time during active disease. We hypothesized that elevated tissue factor pathway inhibitor (TFPI) levels during active inflammation contribute to this paradox. **Methods:** We prospectively enrolled 25 pediatric patients (10 Crohn’s disease [CD], 15 ulcerative colitis [UC]) aged 7–18 years with newly diagnosed IBD. Blood samples were collected at diagnosis and in remission. Thrombin generation was assessed using calibrated automated thrombography. Plasma levels of TFPI, tissue factor activity (TFA), vascular endothelial growth factor (VEGF), and interleukin-6 (IL-6) were measured. **Results:** TFPI levels correlated positively with thrombin generation lag time (r = 0.43, $p_{\mathrm{adj}}$ < 0.05) and disease activity scores (r = 0.54, $p_{\mathrm{adj}}$ < 0.05) in patients with active disease (PCDAI/PUCAI > 0). Longitudinal analysis of 16 patients achieving remission revealed elevated TFPI and prolonged lag time during active disease compared to quiescence (both $p_{\mathrm{adj}}$ < 0.05), while TFA did not change significantly. VEGF decreased significantly upon remission ($p_{\mathrm{adj}}$ < 0.05), whereas IL-6 showed no significant change. **Conclusions:** Elevated TFPI levels during active IBD likely contribute to the paradoxical prolongation of thrombin generation lag time. TFPI normalization upon remission reflects vascular inflammation resolution, suggesting TFPI as a potential biomarker and therapeutic target.

## 1. Introduction

Inflammatory Bowel Disease (IBD) is characterized by chronic inflammation of the digestive tract with active and inactive stages. Most patients with IBD can be classified into either suffering from Crohn’s disease (CD) or ulcerative colitis (UC). CD may affect multiple parts of the whole digestive tract, predominantly the terminal ileum, with a transmural inflammation, while UC affects mainly the endothelial layer of parts of the colon or the entire colon [1].

IBD is gaining more and more importance in pediatric care since studies report increasing numbers of pediatric-onset cases [2,3]. The peak incidence of IBD occurs in patients between the ages of 15 and 30 years [4]. A total of 20% to 25% of all patients are diagnosed before the age of 18 [5,6,7,8]. Disease severity is usually accentuated in children and adolescents with more extensive intestinal involvement [1]. A total of 22% of all pediatric IBD patients exhibit an atypical initial disease presentation with symptoms such as growth failure, anemia, or perianal disease. Mortality is three-fold increased in IBD patients with pediatric-onset in comparison to the normal population [8].

The etiology of IBD is not fully elucidated, but genetic predisposition and an autoimmune response towards healthy intestinal tissue have been discovered [9]. IBD patients also exhibit changes in coagulation that result in a two- to three-fold higher risk for venous thromboembolism compared to the normal population [10], with pediatric patients showing an even higher, approximately six-fold increased risk versus age-matched controls [11]. Inflammation and coagulation crosstalk in a synergistic manner [12]. They enhance each other in a vicious cycle driving the disease. Cytokines released upon chronic inflammation change the phenotype of endothelial cells to a more procoagulant phenotype. The endothelial barrier function and thrombomodulin expression are decreased, while adhesion molecules and tissue factor are over-expressed [9]. Furthermore, cytokines associated with chronic inflammation (e.g., TNF-α, IL-6, IL-β) shift the balance of plasmatic coagulation leading to increased thrombin generation [13].

The increased thrombotic incidence in IBD triggered pathophysiological studies investigating abnormalities in the hemostatic system. In our own studies, we measured in vitro thrombin generation in children with CD using calibrated automated thrombography and showed that all patients exhibited a higher endogenous thrombin potential (ETP) relative to healthy age-matched controls. Thrombin generation parameters correlated with the Pediatric Crohn’s Disease Activity Index (PCDAI). Patients in the active state of CD exhibited a higher ETP [14]. Furthermore, we investigated the levels of circulating procoagulant microparticles and their influence on thrombin generation in children with CD and UC. We found significantly higher microparticle activity in patients with active and inactive CD, and active UC compared with controls, but no significant influence on thrombin generation [15].

When investigating thrombin generation in CD patients, we frequently observed a pattern that was counterintuitive: While ETP and peak thrombin generation were substantially increased in active versus quiescent state, the lag time was longer in the active than in the quiescent state [14]. While this finding did not reach statistical significance in our study, it was reproduced by Stercel et al. in their investigation of the effect of anti-SARS-CoV-2 BNT162b2 mRNA vaccination on thrombin generation in children with IBD [16]. In their cohort of pediatric CD patients, the association between ETP and disease activity (PCDAI) disappeared at follow-up when all CD patients were in remission, yet the lag time parameter showed a significant positive correlation with PCDAI. This consistent observation across independent studies suggests that prolonged lag time may represent a distinct hemostatic signature of active CD, potentially indicating a delayed onset of thrombus formation. Hence, we initiated another study longitudinally investigating thrombin generation in pediatric IBD patients with a special focus on lag time and tissue factor pathway inhibitor, since an increase secondary to endothelial decay may explain this conundrum.

We aimed to determine whether elevated plasma TFPI levels underlie the paradoxical prolongation of thrombin generation lag time in active pediatric IBD and whether TFPI tracks clinical disease activity. We further measured tissue factor activity and inflammatory markers and evaluated the response to exogenous TFPI to assess potential disease-state differences in TFPI sensitivity.

## 2. Results

### 2.1. Baseline Characteristics and Treatment

The study cohort comprised 10 children with CD and 15 children with UC. Baseline characteristics and routine inflammatory and coagulation markers are presented in Table 1. UC patients were, on average, 1.5 years younger than the CD group, with the majority of participants in both groups being adolescents. UC patients exhibited lower CRP and fibrinogen levels than CD patients.

The therapeutic approach for patients was determined by the type and severity of the disease. At baseline, no specific treatment was administered. Induction therapy commonly involved the use of Mesalazine (3/10 [30%] of CD, 14/15 [93%] of UC), and systemic glucocorticoids (3/10 [30%] of CD, 5/15 [33%] of UC). For maintenance therapy, the most frequently prescribed agents were Mesalazine (2/10 [20%] of CD, 11/15 [73%] of UC), Azathioprine (6/10 [60%] of CD, 4/15 [27%] of UC), and Infliximab (5/10 [50%] of CD, 4/15 [27%] of UC) either as monotherapy or in combination. A subset of patients received treatment with Adalimumab, Methotrexate, or Budesonide.

### 2.2. Association of Hemostatic Parameters with Clinical Disease Activity

**Figure 1:**
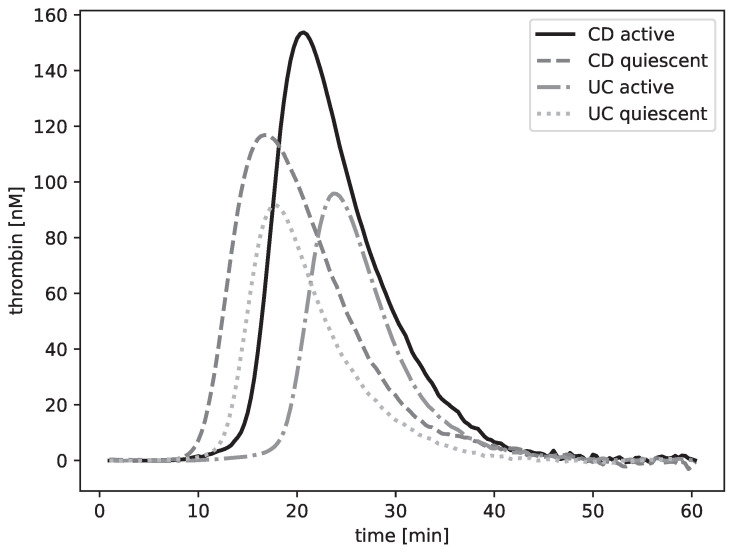
Representative thrombin generation traces of CD and UC patients during active disease and quiescence.

Thrombin generation profiles in CD patients demonstrated a trend toward higher peak values and greater ETP compared to UC patients, though these differences did not reach statistical significance (Peak: *p* = 0.161; ETP: *p* = 0.130; Figure 1, Table 2). Reference measurements in a cohort of young healthy adults analyzed under identical assay conditions (0.5 pM TF) are provided in Supplementary Table S1. Compared to these benchmarks, IBD patients exhibited substantially higher ETP and shorter lag times, consistent with an overall prothrombotic state.

**Figure 2:**
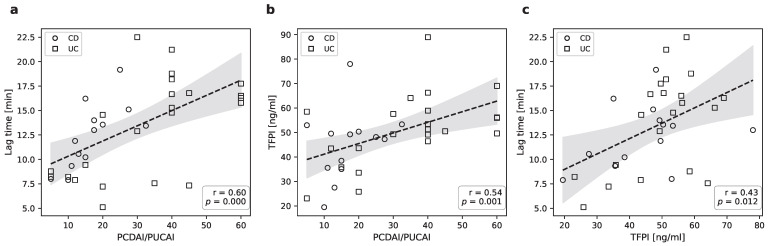
Correlations between clinical disease activity, TFPI and thrombin lag time in IBD patients. Panels (**a**–**c**) show scatterplots of individual patient with visualized linear fit and 95% confidence interval. R and *p* denote Spearman’s rank correlation coefficient (ρ) and the corresponding two-tailed *p*-value, respectively.

To evaluate whether plasma TFPI and thrombin generation parameters track clinical disease severity, we performed Spearman rank correlation analyses with PCDAI/PUCAI scores. To avoid floor effects associated with a disease activity score of zero (clinical remission), correlation analyses were restricted to patients with active disease (PCDAI/PUCAI > 0). Consistent with previous reports [16], a positive correlation was observed between thrombin generation lag time and PCDAI/PUCAI scores (r = 0.60, $p_{\mathrm{adj}}$ < 0.05; Figure 2a). Furthermore, plasma TFPI levels correlated significantly with clinical disease activity (r = 0.54, $p_{\mathrm{adj}}$ < 0.05; Figure 2b). Crucially, we identified a robust positive correlation between plasma TFPI levels and the thrombin generation lag time (r = 0.43, $p_{\mathrm{adj}}$ < 0.05; Figure 2c). To assess the independence of this relationship, exploratory multivariable inferential analyses were performed. In these parsimonious models, TFPI remained associated with lag time after adjustment for IL-6 (β = 0.14, *p* = 0.041), whereas this association was attenuated in models including VEGF or TFA, likely reflecting limited statistical power and the biological overlap of endothelial activation markers.

Similarly, VEGF and IL-6 showed a positive correlation with PCDAI/PUCAI scores (r = 0.46, *p* < 0.05 and r = 0.46, *p* < 0.05, respectively), whereas TFA did not (r = 0.16, *p* = 0.488). While the correlations for VEGF, and IL-6 reached nominal significance, they should be interpreted as exploratory trends as they did not survive stringent FDR correction across all parameters.

### 2.3. Longitudinal Comparison of Active and Quiescent Disease States

**Figure 3:**
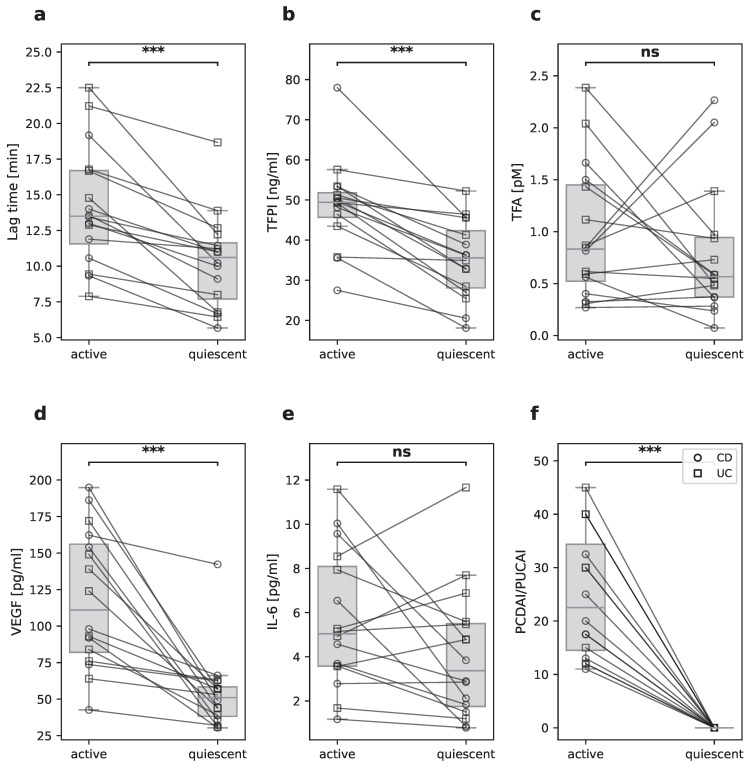
Paired analysis of 16 pediatric patients (CD, *n* = 8; UC, *n* = 8) during active and quiescent disease states. (**a**) Thrombin generation lag time, (**b**) TFPI, (**c**) TFA, (**d**) VEGF, (**e**) IL-6, and (**f**) PCDAI/PUCAI scores. Individual data points are represented by circles for CD patients and squares for UC patients, with connecting lines indicating longitudinal follow-up. *** *p* < 0.001 for two-sided Wilcoxon signed-rank test.

To provide a more robust, within-patient confirmation of these associations and to account for individual baseline variability, we performed a longitudinal analysis on 16 patients (CD, *n* = 8; UC, *n* = 8) who presented with significant disease activity (PCDAI/PUCAI > 10) and achieved complete remission (PCDAI/PUCAI = 0) during the study period (Figure 3f). The median time from active disease to remission was 105 days (IQR 50–226 days). The remaining patients were excluded due to insufficient baseline disease activity or failure to achieve remission.

Standard coagulation parameters remained stable across disease states. APTT did not differ significantly between active and quiescent disease in CD patients (median: 30.90 [IQR 27.15–31.65] vs. 32.00 [IQR 29.80–33.75] s, $p_{\mathrm{adj}}$ = 0.592) or UC patients (median: 31.00 [IQR 30.80–34.42] vs. 32.60 [IQR 30.95–33.52] s, $p_{\mathrm{adj}}$ = 0.703). Similarly, PT showed no significant changes in CD (median: 96.0 [IQR 92.5–114.0] vs. 93.0 [IQR 90.5–102.5]%, $p_{\mathrm{adj}}$ = 0.592) or UC patients (median: 94.5 [IQR 87.0–100.5] vs. 92.5 [IQR 84.8–111.3]%, $p_{\mathrm{adj}}$ = 0.592).

However, in agreement with the correlation analyses, both lag time (Table 2) and plasma TFPI levels were higher during active disease compared to the quiescent stage (Figure 3a,b). TFPI levels decreased from a median of 49.4 ng/mL [IQR 45.0–51.1] to 34.6 ng/mL [IQR 25.4–36.9] in CD patients, and from 49.9 ng/mL [IQR 45.7–51.8] to 38.1 ng/mL [IQR 31.7–45.8] in UC patients ($p_{\mathrm{adj}}$ = 0.034 for both groups). Interestingly, TFA levels showed no statistically significant change between active and quiescent disease (CD: median 0.69 [IQR 0.38–1.01] vs. 0.37 [IQR 0.27–0.95] pg/mL, $p_{\mathrm{adj}}$ = 0.945; UC: median 0.99 [IQR 0.61–1.58] vs. 0.66 [IQR 0.54–0.94] pg/mL, $p_{\mathrm{adj}}$ = 0.592; Figure 3c). IL-6, a marker of systemic inflammation, also showed no significant change following disease remission in either CD (median 4.13 [IQR 3.38–7.30] vs. 1.98 [IQR 1.34–2.87] pg/mL, $p_{\mathrm{adj}}$ = 0.060) or UC patients (median 5.21 [IQR 4.59–8.09] vs. 5.53 [IQR 4.79–7.08] pg/mL, $p_{\mathrm{adj}}$ = 0.765; Figure 3e). Given the limited sample size, these findings should not be interpreted as evidence for absence of an effect. In contrast, VEGF, a marker of vascular inflammation, decreased substantially in both groups (CD: median 126.0 [IQR 87.4–168.2] vs. 53.1 [IQR 34.9–63.4] pg/mL, $p_{\mathrm{adj}}$ = 0.034; UC: median 108.5 [IQR 82.0–141.5] vs. 48.5 [IQR 42.8–57.0] pg/mL, $p_{\mathrm{adj}}$ = 0.034; Figure 3d).

To explore potential confounding by treatment, patients were stratified into those receiving biologic therapy and those receiving non-biologic or no systemic therapy. The reduction in both plasma TFPI levels and thrombin generation lag time upon remission was observed with 100% consistency in patients receiving biologic therapy as well as in those on non-biologic regimens (Supplementary Table S3). Furthermore, cross-sectional correlations between disease activity, TFPI, and lag time remained directionally consistent across both treatment strata (Supplementary Table S2).

Changes in thrombin generation parameters following addition of exogenous TFPI showed no significant differences between CD and UC patients or between active and quiescent disease stages (Table 3), suggesting that an altered sensitivity of the hemostatic system to TFPI plays no part in the observed changes in lag time.

## 3. Discussion

We and others have observed that pediatric patients with IBD exhibit a paradoxical hemostatic profile: higher disease activity is associated with prolonged lag time of thrombin generation despite increased overall thrombin generation [14,16]. This kinetic dissociation does not imply hypocoagulability; while elevated TFPI delays the initiation phase, the propagation phase remains markedly enhanced, as evidenced by increased ETP and peak thrombin. Once triggered, thrombin generation proceeds more robustly, resulting in a net hypercoagulable state and increased thrombotic risk despite the delayed onset. This phenomenon is only detectable through thrombin generation assays, as standard coagulation parameters (aPTT and PT) lack sensitivity to variations in endogenous coagulation inhibitors [17]. Using low concentrations of TF in thrombin generation assays, we reproduced this finding: in both CD and UC patients, ETP was elevated while lag time was prolonged during active disease compared to remission. Since lag time is primarily determined by the TF/TFPI balance, we hypothesized that elevated TFPI levels contribute to this prolongation during active inflammation.

This hypothesis is supported by a significant triangulation between clinical disease activity, plasma TFPI levels, and thrombin generation lag time observed in our cohort. TFPI concentrations were elevated above the pediatric reference range [18] during active CD and UC and normalized upon remission, paralleling the behavior of lag time. In addition, both TFPI levels and lag time were significantly associated with disease activity scores, indicating that these parameters reflect the inflammatory state.

Reference data from a benchmark cohort of young healthy adults (Supplementary Table S1) provide further context for these alterations. TFPI levels in this group were consistent with published reference ranges [18], further validating that the elevations seen in active IBD are pathological. Interestingly, these healthy controls exhibited substantially lower ETP and longer lag times than IBD patients despite having lower TFPI levels. This observation reflects the profound prothrombotic shift in IBD, where baseline coagulation pre-activation accelerates thrombin initiation beyond healthy physiological levels. While thrombin generation is age-dependent, these fundamental differences cannot be explained by age alone, as the magnitude of pathological change in IBD far exceeds expected physiological variation [19].

However, increased TFPI levels could theoretically be counteracted in vivo by equivalent increases in TF levels. We therefore measured TFA longitudinally but found no significant difference between active disease and remission, suggesting that the TFPI/TF balance is indeed elevated during active inflammation.

Alternatively, the observed prolongation of lag time could result from altered plasma sensitivity to TFPI. Potential endogenous modulators of TFPI activity include autoantibodies, polyphosphates, neutrophil extracellular traps, and lipoproteins [20,21]. However, when we added exogenous TFPI to thrombin generation assays, the response was comparable between active disease and remission, suggesting that the observed changes in lag time are primarily related to varying TFPI concentrations, as no evidence of an altered sensitivity to TFPI activity was found.

TFPI levels in adult IBD have yielded inconsistent results and variable correlations with inflammatory markers [22,23]. In our pediatric cohort, VEGF decreased substantially upon remission, whereas IL-6 showed only modest, non-significant downward trends. While the modest sample size and inherent variability of circulating cytokines preclude definitive conclusions regarding “resolution kinetics,” the observed pattern is compatible with differential regulation of endothelial and systemic inflammatory pathways. Specifically, elevated TFPI—a known marker of endothelial activation [24]—and its decline in remission align with a vascular-inflammatory function. In contrast, the relative stability of IL-6 may reflect limited power, but could also suggest that some systemic inflammatory signaling persists, potentially contributing to sustained hepatic TF production and TFA levels despite clinical improvement.

In this context, we focused on biologic therapy as a primary differentiator because anti-TNF agents are known to directly modulate endothelial activation [25], potentially affecting the dynamic release of TFPI into the circulation. However, our exploratory stratification by biologic versus non-biologic therapy did not provide robust evidence for a treatment-specific pattern in the associations between disease activity, TFPI, and thrombin generation parameters. While the uniform reduction in TFPI and lag time across both strata (Supplementary Table S3) supports disease resolution as a consistent driver, any apparent numerical differences between groups must be interpreted cautiously. Given the limited sample size and the frequency of overlapping treatment regimens, these findings remain exploratory.

TFPI may not merely serve as a marker of endothelial damage relevant for coagulation, but rather plays an integral role in the inflammatory pathophysiology of IBD. He et al. demonstrated that TFPI expression is significantly increased in UC tissue and correlates with disease activity [26]. Critically, TFPI functions beyond its anticoagulant properties: it acts as an indirect inhibitor of matrix metalloproteinases (MMPs) in the extracellular matrix [27], which are markedly upregulated in inflamed IBD mucosa and contribute to tissue destruction. Additionally, TFPI suppresses inflammatory mediator release and inhibits activation of inflammatory cells [28]. This multifunctional profile suggests that TFPI is an active participant in the processes underlying both thrombotic risk and tissue inflammation, rather than simply a passive biomarker of vascular injury. However, given its essential anticoagulant function, any potential therapeutic modulation of this pathway would require careful evaluation to avoid exacerbating thrombotic tendencies.

Our study has several limitations. First, the cohort size was relatively small, with 25 patients for cross-sectional correlation analyses and 16 for longitudinal comparisons—a common challenge in pediatric research. While the primary longitudinal findings for TFPI and lag time were robust and survived multiple testing correction, the study was not formally powered a priori to detect modest effects. Consequently, the non-significant findings for parameters such as TFA and IL-6, particularly in disease-specific subgroups, should be interpreted as inconclusive rather than as evidence of an absence of effect. Second, we did not directly measure tissue TFPI which would further clarify TFPI’s dual role in coagulation and inflammation. Third, the study lacked an age-matched pediatric control group; although reference data from young healthy adults measured under identical conditions were provided, residual effects of age cannot be fully excluded. Finally, although exploratory stratification by treatment type suggests that the hemostatic shifts were consistent across therapeutic groups, the small sample size and frequent use of combination therapies preclude a definitive assessment of drug-specific effects. Consequently, the observed changes in TFPI and thrombin generation reflect an overall clinical response, and residual confounding by specific therapeutic agents cannot be fully excluded. Importantly, the study design does not allow causal inference, as TFPI was not directly manipulated; thus, whether reducing TFPI levels would normalize thrombin generation lag time remains to be determined in future functional studies.

In conclusion, our findings suggest that prolonged thrombin generation lag time in active pediatric IBD is associated with elevated plasma TFPI levels, likely reflecting the state of vascular inflammation. While TFPI and VEGF concentrations were significantly lower during clinical remission—consistent with a resolution of vascular inflammatory activation—TFA and IL-6 levels remained relatively stable in this cohort. This divergence may indicate that distinct components of the inflammatory and hemostatic systems are differentially regulated or vary in their baseline variability. Our findings reveal a novel aspect of IBD pathophysiology with implications for both thrombotic risk stratification and understanding inflammatory progression in these patients. The dual role of TFPI as both an anticoagulant and anti-inflammatory mediator warrants further investigation as a component of the vascular inflammatory response in IBD and as a potential biomarker linking coagulation and inflammation.

## 4. Materials and Methods

### 4.1. Participants and Preanalytics

This prospective longitudinal cohort study was conducted in accordance with the Declaration of Helsinki and approved by the Ethics Committee of the Medical University of Graz, Austria (EK-No. 32-019 ex 19/20). Written informed consent was obtained from all legal guardians and, when age-appropriate, from the participants themselves.

Patients aged 7–18 years who were admitted between 30 October 2019, and 29 October 2022, to the Department of Pediatrics and Adolescent Medicine at the Medical University of Graz with suspected inflammatory bowel disease (IBD) were recruited for this study. Patients typically presented with characteristic gastrointestinal symptoms including hematochezia, diarrhea, and abdominal pain. Diagnostic evaluation included esophagogastroduodenoscopy (EGD) and ileocolonoscopy.

Patients with newly diagnosed ulcerative colitis (UC) or Crohn’s disease (CD) at disease onset were included. Diagnosis was established according to ECCO/ESPGHAN criteria through a combination of typical clinical presentation, characteristic endoscopic findings, histopathological examination, and imaging studies. Disease activity was assessed at each blood sampling timepoint using the Pediatric Ulcerative Colitis Activity Index (PUCAI) for UC patients and the Pediatric Crohn’s Disease Activity Index (PCDAI) for CD patients [29]. Patients were excluded if they had pre-existing medical conditions, hereditary or acquired coagulation disorders, or disease relapses. Use of anticoagulant medications including heparins (enoxaparin, dalteparin) or factor Xa inhibitors (edoxaban, rivaroxaban) was an exclusion criterion.

Upon admission, venous blood samples were collected without application of venostasis in pre-citrated S-Monovette® tubes (Sarstedt, Nümbrecht, Germany) containing 0.106 mol/L trisodium citrate solution. Samples were centrifuged twice at 2400× *g* for 10 min to obtain platelet-poor plasma, which was then aliquoted and stored at −80 °C until analysis. Blood samples were collected at disease onset (at diagnosis), during treatment (3–6 weeks after starting medication), and during remission (defined as PUCAI/PCDAI < 10).

To provide physiological context for the assay parameters, a separate benchmark cohort of young healthy adults was analyzed under identical experimental conditions (see Supplementary Table S1).

### 4.2. Calibrated Automated Thrombography

Calibrated automated thrombography (CAT) was performed on plasma samples from IBD patients as previously described [30]. Prior to analysis, samples were centrifuged at 11,000× *g* for 20 min to remove residual cell fragments. Thrombin generation was initiated using a low tissue factor concentration (0.5 pM) in the presence of 4 µM phospholipids to maximize sensitivity to endogenous TFPI levels. Thrombin generation curves were recorded and analyzed using Thrombinoscope software (v12.0, Diagnostica Stago, Asnières-sur-Seine, France). The intra-assay CV for these parameters was 4.7%, and the inter-assay CV was 7.3%. To minimize analytical variability, all longitudinal samples from each patient were analyzed within the same assay run. To assess the sensitivity of plasma to TFPI inhibition, measurements were repeated following addition of exogenous TFPI (50 ng/mL; Abcam, Cambridge, UK). Preliminary experiments demonstrated that the inhibitory effect of TFPI followed a linear dose-dependent relationship with the absolute change in lag time (ΔLag time), but functioned independently of the patient’s baseline lag time.

### 4.3. Specific Assays and Laboratory Parameters

TF procoagulant activity was assessed using a chromogenic assay (ACTICHROME® TF, BioMedica Diagnostics, Stamford, CT, USA). TFPI concentrations were determined using a sandwich ELISA (Quantikine™, DTFP10, R&D Systems, Minneapolis, MN, USA). Human vascular endothelial growth factor (VEGF) and interleukin-6 (IL-6) were measured using sandwich SimpleStep ELISA kits (Abcam, Cambridge, UK). All assays were performed according to the manufacturers’ instructions.

Standard clinical laboratory parameters—including fibrinogen, prothrombin time (PT), activated partial thromboplastin time (aPTT), C-reactive protein (CRP), and fecal calprotectin—were analyzed according to established institutional protocols.

### 4.4. Statistics

Statistical analyses were performed using Python 3.11.7 with the SciPy (v2.4.1) and NumPy (v1.17.0) libraries. Continuous variables are presented as median with interquartile range (IQR). Baseline characteristics and biomarker levels between CD and UC groups were compared using the Mann–Whitney U test. For longitudinal comparisons between active disease and remission states within the same patients, the Wilcoxon signed-rank test was applied. Correlations between continuous variables (e.g., TFPI levels, lag time, and PCDAI/PUCAI scores) were assessed using Spearman’s rank correlation coefficient (r). Additionally, parsimonious multivariable linear regression was used for exploratory inferential analysis of TFPI independence; due to the limited sample size, these models were restricted to two predictors to minimize the risk of overfitting. For all inferential analyses, *p*-values were adjusted for multiple testing using the Benjamini-Hochberg false discovery rate (FDR) procedure to account for the number of simultaneous hypotheses tested. Statistical significance was defined as an adjusted *p* < 0.05.

For the primary longitudinal endpoint (within-patient change in thrombin generation lag time from active disease to remission), sample size estimation for a paired comparison assumed a mean difference of 3.0 min and an SD of paired differences of 2.5 min ($d_{z}$ = 1.20). With two-sided α = 0.05 and 80% power, this corresponds to a required sample size of eight paired patients.

## Figures and Tables

**Table 1:** Baseline characteristics and treatments of pediatric IBD patients at first visit.

	CD (*n* = 10)	UC (*n* = 15)
Age [years]	16.3 (15.1–17.1)	14.9 (13.7–15.8)
Male/Female [*n*]	5/5	9/6
Weight [kg]	46.4 (25.5–60.0)	49.8 (49.3–63.3)
PCDAI/PUCAI	16.3 (13.5–19.4)	40 (15–40)
Calprotectin [µg/g]	610 (190–844)	624 (125–928)
CRP [mg/dL]	20.1 (10.4–30.9)	1.9 (1.1–11.9)
Fibrinogen [mg/dL]	442 (354–485)	327 (267–344)
PT [%]	96 (89–113)	95 (87–101)
aPTT [s]	30.1 (26.9–30.9)	31.0 (30.7–35.1)

Data are presented as median (interquartile range). CD, Crohn’s disease; UC, ulcerative colitis; PCDAI, Pediatric Crohn’s Disease Activity Index; PUCAI, Pediatric Ulcerative Colitis Activity Index; CRP, C-reactive protein; PT, prothrombin time; aPTT, activated partial thromboplastin time.

**Table 2:** Thrombin generation parameters in active and quiescent inflammatory bowel disease.

	CD Active	CD Quiescent	$p_{\mathrm{adj}}$	UC Active	UC Quiescent	$p_{\mathrm{adj}}$
Lag time [min]	13.22 (11.56–13.67)	10.11 (8.50–11.06)	**0.034**	15.73 (12.03–17.89)	11.61 (7.70–12.98)	**0.034**
Time to peak [min]	16.45 (15.75–17.72)	14.84 (12.78–15.75)	0.354	20.11 (16.98–22.53)	16.06 (13.72–17.25)	**0.034**
Peak [nM]	167 (97–235)	110 (66–173)	0.266	91 (82–131)	86 (74–102)	0.592
ETP [nM·min]	1389 (1093–1631)	1102 (712–1483)	0.266	1038 (854–1139)	933 (922–985)	0.641

Data are presented as median (interquartile range). Statistical comparisons were performed using Wilcoxon signed-rank test between active and quiescent disease states within each disease group. *p*-values were adjusted for multiple testing using the Benjamini–Hochberg procedure. **Bold** values indicate statistical significance ($p_{\mathrm{adj}}$ < 0.05). CD, Crohn’s disease; UC, ulcerative colitis; ETP, endogenous thrombin potential.

**Table 3:** Effect of exogenous TFPI on thrombin generation parameters in active and quiescent inflammatory bowel disease.

	CD Active	CD Quiescent	$p_{\mathrm{adj}}$	UC Active	UC Quiescent	$p_{\mathrm{adj}}$
ΔLag time [min]	1.94 (0.56–4.12)	1.44 (1.03–3.25)	0.641	1.55 (0.92–2.21)	1.11 (0.53–1.81)	0.641
ΔTime to peak [min]	2.39 (0.44–4.00)	1.39 (1.12–3.31)	0.641	1.44 (0.69–2.36)	1.05 (0.39–1.82)	0.703
ΔPeak [nM]	1.59 (−17.05–9.40)	−6.14 (−19.60–3.37)	0.641	−1.68 (−3.81–6.77)	−4.27 (−13.31–4.54)	0.641
ΔETP [nM·min]	−20.9 (−63.3–59.8)	−51.03 (−110.06–−10.61)	0.592	−7.68 (−34.43–7.63)	−43.95 (−97.64–6.17)	0.765

Data are presented as median Δ (with TFPI − without TFPI) (IQR). Statistical comparisons were performed using Wilcoxon signed-rank test between active and quiescent disease states within each disease group. *p*-values were adjusted for multiple testing using the Benjamini-Hochberg procedure. CD, Crohn’s disease; UC, ulcerative colitis; ETP, endogenous thrombin potential; TFPI, tissue factor pathway inhibitor.

## Data Availability

The data presented in this study are available on request from the corresponding author due to stringent data protection legislation governing sensitive pediatric patient data.

## Supplementary Materials

The following supporting information can be downloaded at: https://www.mdpi.com/article/10.3390/ph19050738/s1, Table S1: Thrombin generation parameters and TFPI levels in a benchmark cohort of healthy young adults; Table S2: Treatment-stratified correlations between clinical disease activity, TFPI, and thrombin generation parameters in pediatric IBD; Table S3: Treatment-stratified longitudinal changes from active disease to remission.
