# Supplementary material for: Plasma Tissue Factor Pathway Inhibitor Levels Correlate with Disease Activity and Are Associated with Altered Thrombin Generation in Pediatric Inflammatory Bowel Disease
Source: Pharmaceuticals (Basel). 2026 May 8;19(5):738. doi: 10.3390/ph19050738 (PMC13210234; doi:10.3390/ph19050738)
Supplement: Supplementary file 1 [file pharmaceuticals-19-00738-s001.zip › pharmaceuticals-4295962-supplementary.pdf]

Table S1: Thrombin generation parameters and TFPI levels in a benchmark cohort of healthy young adults.

**Age [years]**	23.0 (20.5-26.5)
**Male/Female [N]**	11/12
**Lag time [min]**	17.11 (15.27–21.46)
**Time to peak [min]**	21.46 (19.86–27.16)
**Peak [nM]**	43 (27–50)
**ETP [nM*min]**	440 (329–521)
**ΔLag time [min]**	1.53 (0.70–3.48)
**ΔTime to peak [min]**	2.17 (0.83–5.09)
**ΔPeak [nM]**	-4.46 (-11.64–2.51)
**ΔETP [nM*min]**	-39.72 (-124.30–-5.42)
**TFPI [ng/ml]**	27.8 (26.4–30.7)

Data are presented as median (interquartile range). Thrombin generation was measured using 0.5 pM tissue factor and 4 µM phospholipids to ensure comparability with the study cohort. Δ parameters represent the absolute change following the addition of exogenous TFPI (50 ng/mL). ETP, endogenous thrombin potential; TFPI, tissue factor pathway inhibitor.

Table S2: Treatment-stratified correlations between clinical disease activity, TFPI, and thrombin generation parameters in pediatric IBD

**Analysis**	**Biologic (N=10)**	**Non-biologic (N=15)**
**PCDAI/PUCAI vs Lag time**	r=0.66, p=0.001	r=0.40, p=0.125
**PCDAI/PUCAI vs TFPI**	r=0.50, p=0.031	r=0.57, p=0.017
**TFPI vs Lag time**	r=0.17, p=0.482	r=0.73, p=0.002

Spearman rank correlation coefficients (ρ) and corresponding two-tailed p-values for the associations between clinical disease activity (PCDAI/PUCAI), plasma TFPI levels, and thrombin generation lag time, stratified by treatment group (biologic vs. non-biologic). Analyses were restricted to patients with active disease (PCDAI/PUCAI > 0). The biologic group included patients receiving anti-TNF or anti-integrin therapy (e.g., infliximab, adalimumab, vedolizumab), whereas the non-biologic group comprised patients receiving conventional therapy or no systemic treatment. Due to small subgroup sizes and overlapping combination therapies, these analyses are exploratory and not intended for formal between-group comparison.

Table S3: Treatment-stratified longitudinal changes from active disease to remission

**Parameter**	**Biologic (n=8)**	**Non-biologic (n=8)**
**Lag time [min] (active → remission)**	−4.17 (−8.24 to −3.50)	−2.22 (−2.92 to −1.45)
**TFPI [ng/mL] (active → remission)**	−15.95 (−18.27 to −10.66)	−11.63 (−16.49 to −4.42)
**TFA [pM] (active → remission)**	−0.08 (−0.59 to 0.23)	−0.12 (−0.99 to 0.08)
**VEGF [pg/mL] (active → remission)**	−53.30 (−91.04 to −28.84)	−58.00 (−120.50 to −12.40)
**IL-6 [pg/mL] (active → remission)**	−1.76 (−3.19 to −0.27)	−0.08 (−3.26 to 1.31)
**Direction consistent (↓)**	8/8 (100%)	8/8 (100%)

Data are presented as median Δ (IQR). Δ was calculated as remission minus active disease; negative values indicate a decrease during follow-up. The biologic group includes patients receiving anti-TNF or anti-integrin therapy, irrespective of concomitant treatments. The non-biologic group includes patients without biologic exposure. Direction consistency refers to concordant decreases in both TFPI and thrombin generation lag time within individual patients.
